# Limited shifts in the distribution of migratory bird breeding habitat density in response to future changes in climate

**DOI:** 10.1002/eap.2428

**Published:** 2021-08-30

**Authors:** Owen P. McKenna, David M. Mushet, Samuel R. Kucia, Elyssa C. McCulloch‐Huseby

**Affiliations:** ^1^ U.S. Geological Survey Northern Prairie Wildlife Research Center 8711 37th Street SE Jamestown North Dakota 58401 USA

**Keywords:** climate change, migratory bird habitat, North American Prairie Pothole Region, PHyLiSS model, wetland hydrology, wetland management

## Abstract

Grasslands, and the depressional wetlands that exist throughout them, are endangered ecosystems that face both climate and land‐use change pressures. Tens of millions of dollars are invested annually to manage the existing fragments of these ecosystems to serve as critical breeding habitat for migratory birds. The North American Prairie Pothole Region (PPR) contains millions of depressional wetlands that produce between 50% and 80% of the continent’s waterfowl population. Previous modeling efforts suggested that climate change would result in a shift of suitable waterfowl breeding habitat from the central to the southeast portion of the PPR, an area where over half of the depressional wetlands have been drained. The implications of these projections suggest a massive investment in wetland restoration in the southeastern PPR would be needed to sustain waterfowl populations at harvestable levels. We revisited these modeled results indicating how future climate may impact the distribution of waterfowl‐breeding habitat using up‐to‐date climate model projections and a newly developed model for simulating prairie‐pothole wetland hydrology. We also presented changes to the number of “May ponds,” a metric used by the U.S. Fish and Wildlife Service to estimate waterfowl breeding populations and establish harvest regulations. Based on the output of 32 climate models and two emission scenarios, we found no evidence that the distribution of May ponds would shift in the future. However, our results projected a 12% decrease to 1% increase in May pond numbers when comparing the most recent climate period (1989–2018) to the end of the 21st century (2070–2099). When combined, our results suggest areas in the PPR that currently support the highest densities of intact wetland basins, and thus support the largest numbers of breeding‐duck pairs, will likely also be the places most critical to maintaining continental waterfowl populations in an uncertain future.

## Introduction

Wetlands cover 6% of the Earth’s surface (Junk et al. 2013). These ecosystems provide many unique services to society, including enhancing water quality (Verhoeven et al. 2006), storing one‐third of the world's organic soil‐carbon pool (Bernal and Mitsch 2012), recharging groundwater reservoirs (Gurdak and Roe 2010), and providing critical habitat for many different species of migratory birds (Tiner 1984) and many other taxa across all trophic levels (Dudgeon et al. 2006). Freshwater wetlands, especially non‐floodplain depressional wetlands, are particularly sensitive to climatic shifts (Hayashi et al. 2016, McKenna et al. 2017). The biological functioning of these ecosystems is largely dependent on the balance between precipitation inputs and evapotranspiration demands, which makes them extremely sensitive to changes in precipitation and temperature (Taylor et al. 2013).

Non‐floodplain depressional wetlands generally exist in semi‐arid and arid biomes with high rates of land‐use and land‐cover change, both of which can directly and indirectly impact ecosystem functioning (Scanlon et al. 2005, Creed et al. 2017). Limited protections for non‐floodplain wetlands (Golden et al. 2017) make them vulnerable to drainage (Dahl 2014) and agricultural conversion (Samson and Knopf 1994), which can be exacerbated by changes in climate (McKenna et al. 2019).

In North America, millions of non‐floodplain wetlands known as prairie potholes occupy glacially formed depressions that collectively make up the 10th largest (˜800,000 km^2^) wetland ecosystem complex in the world (Keddy et al. 2009). The North American Prairie Pothole Region (PPR) is a continentally significant area for biodiversity (Ando and Mallory 2012) and the wetlands within the region provide more breeding habitat for waterbirds than any other ecosystem in North America (Dahl 2014). Precipitation and temperature driven changes in these wetlands determine the availability of suitable habitat for waterbirds (Haig et al. 2019, Mushet et al. 2020). However, the wetland ecosystems of the PPR are disappearing (Calhoun et al. 2017) due to agricultural intensification (Anteau et al. 2016, McKenna et al. 2019) and high regional climatic variability (Winter and Rosenberry 1998).

The disproportionate importance of the PPR for a large number of species and the vulnerability of prairie‐pothole wetlands to climate and land‐use change has led to tens of millions of dollars being spent annually in the PPR to fund habitat‐management programs aimed at the conservation of waterbird species (Mattsson et al. 2020). Additionally, multiple wetland monitoring efforts have been sustained for over three decades and enabled the development of different modeling tools and approaches to assess how climate change could impact wetlands and waterbirds in the PPR (Johnson and Poiani 2016, McIntyre et al. 2019, McKenna et al. 2019). Despite past efforts to forecast the future impact that climate change may have on prairie‐pothole wetlands, the conservation and management community has identified the need for more robust and relevant climate/wetland projections (Rushing et al. 2020) using climate‐modeling best practices (Sofaer et al. 2017) for managing this ecosystem of continental and global importance (Yocum and Ray 2019).

Our research objective was to improve on current projections of the impact that climate change will have on the future suitability of waterfowl habitat in the PPR. We designed this study using advanced mechanistic modeling tools, the most up‐to‐date climate data, best‐practice climate modeling procedures, and management‐relevant metrics to specifically address this conservation and decision‐making need. Specifically, the aim of our study was to answer two questions related to this objective. First, how are precipitation and temperature projected to change throughout the PPR under the most recent global‐climate‐model (GCM) projections? Second, how will the number and spatial distribution of prairie‐pothole wetlands that are ponded during waterfowl breeding season, an index of waterfowl population size, change in the future due to changes in temperature and precipitation?

## Methods

To address our two research questions, we first analyzed downscaled precipitation and temperature data from a suite of the most up‐to‐date GCMs and compared those changes to the last 30 yr of observed precipitation and temperature data to determine a range of projected changes in climate throughout the PPR. We then input those modeled precipitation and temperature data into a mechanistic prairie‐pothole wetland hydrology model to simulate water levels in representative wetlands throughout the six ecoregions of the PPR. Finally, we developed relationships between our mechanistic model output and regional estimates of available waterfowl habitat in order to scale‐up our findings to the whole PPR.

Our work differed from past climate‐change modeling of prairie‐pothole wetlands in three ways. First, we simulated water levels using the Pothole Hydrology‐Linked Systems Simulator (PHyLiSS) model (McKenna et al. 2018) that was designed to accommodate a wide range of wetland and catchment characteristics. Rather than applying different climate‐change manipulations on the same wetland complex as in Johnson et al. (2005), we parameterized each wetland configuration in PHyLiSS using ecoregion‐specific wetland and catchment morphology data from Gleason and Tangen (2008).

Second, we used the currently accepted best 32 GCMs that are part of the coupled model intercomparison project phase 5 (CMIP5) (Taylor et al. 2012) to determine a range of future temperature and precipitation possibilities. In total, we modeled wetland water levels for each of the 32 different GCMs using two different emission scenarios (RCP 4.5 and RCP 8.5). We applied the delta method for evaluating changes between observed precipitation and temperature of the most recent 30‐yr climate period and future GCM projections (Sofaer et al. 2016).

**Fig. 1:**
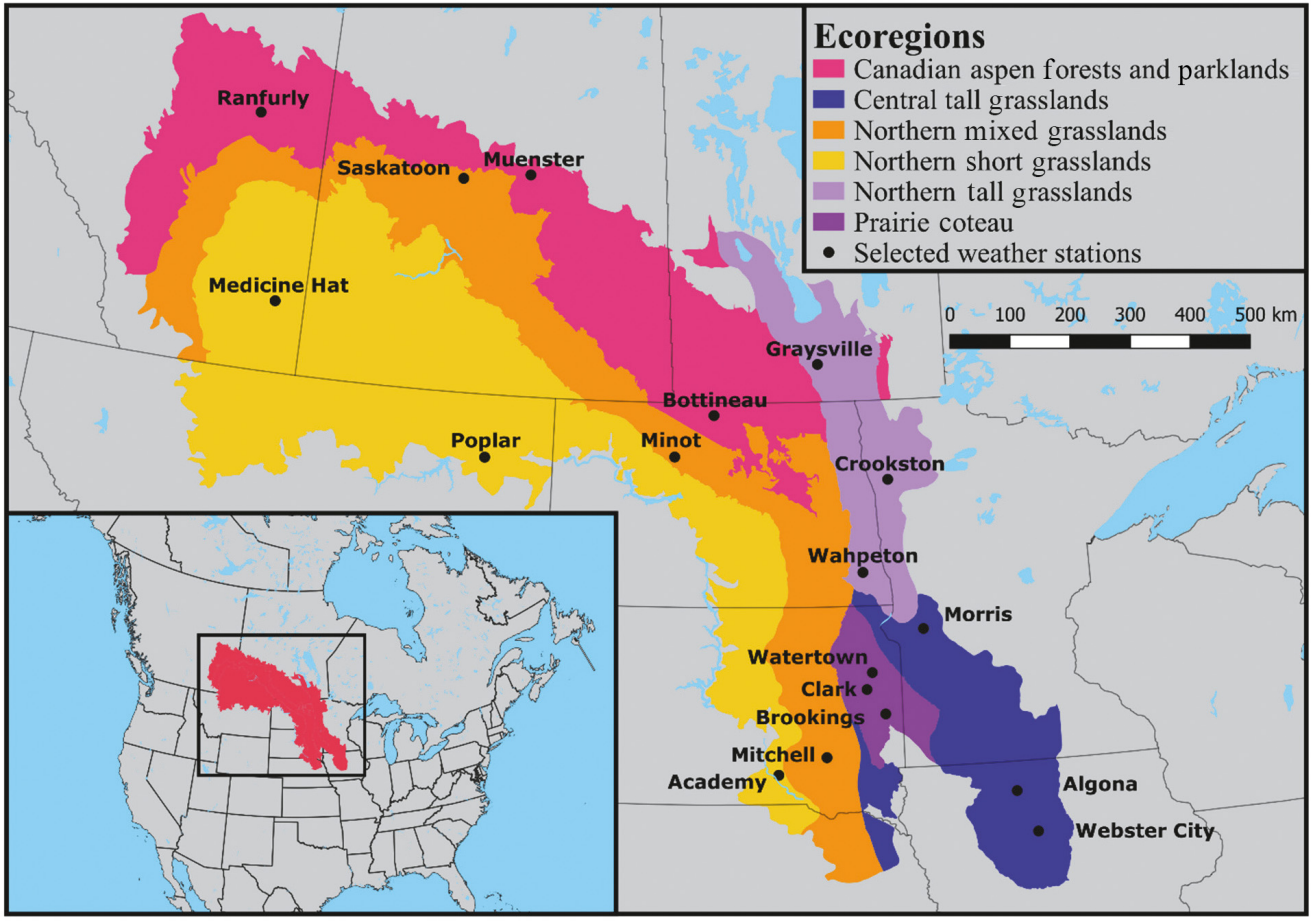
Map of the North American Prairie Pothole Region with ecoregions color coordinated and selected weather stations highlighted as black points. Ecoregion spatial layers provided by Bruce Millett.

Last, we followed the methodological approach for scaling up wetland simulations to regional estimate of ponded wetlands from McKenna et al. (2021). We developed linear regression models for estimating water levels in wetlands during May, which is a critical month for breeding waterfowl. We simulated water pool volumes in both our soil water and ponded water components of wetland basins (hereafter, pool volumes) using PHyLiSS and related those volumes to the corresponding sub‐regional estimates of wetland basins ponded in the month of May (1982–2015). Counts of ponds in the PPR are annually conducted by U.S. Fish and Wildlife Service (USFWS) and subdivided into “strata” polygons (Dahl 2014). These empirically derived “May pond counts” have formed the basis of much prior work on waterfowl population trends in the PPR (Niemuth and Solberg 2003). For comparative purposes, we downscaled climate effects to the same 18 sites across six ecoregions, three sites per ecoregion (Fig. 1) from Johnson and Poiani (2016) as a foundation for our modeling approach to simulate climate change throughout the PPR. We also converted the May pond estimate for each sample stratum to a density by dividing the value by the area of each stratum. We then developed our linear regression models for predicting May ponds using the Annual May pond density for the sample stratum where each of the study sites resides (Appendix S1: Fig. S1). Unless otherwise specified, all analysis and visualization was conducted using R Version 3.6.1 (R Core Team 2017).

### Study site

The North American PPR covers approximately 770,000 km^2^ of the United States and Canada (Fig. 1; Smith et al. 1964). The advance and retreat of the Laurentide ice sheet (Dyke and Prest 1987) resulted in the PPR being covered with low‐permeability glacial till and millions of closed‐catchment depressions (Goldhaber et al. 2014) that are commonly referred to as prairie potholes. The PPR supports 50% to 80% of the continent’s breeding duck population (Batt et al. 1989). In total, almost 120 species of wetland‐dependent migratory birds from 21 different taxonomic families are estimated to utilize wetland habitat in the PPR during their migrations (Steen et al. 2016). The functioning of these critical ecosystems is largely dependent on the balance between atmospheric water inputs and demands, making them extremely sensitive to changes in climate that impact precipitation and temperature (Taylor et al. 2013). Modeling efforts by Johnson et al. (2005) suggested that climate change could limit the viable waterfowl habitat in the PPR to a small area in Iowa, USA that has undergone ˜90% of wetland drainage (Van Meter and Basu 2015, Skopec and Evelsizer 2018).

### Evaluating future changes in climate across the PPR

**Fig. 2:**
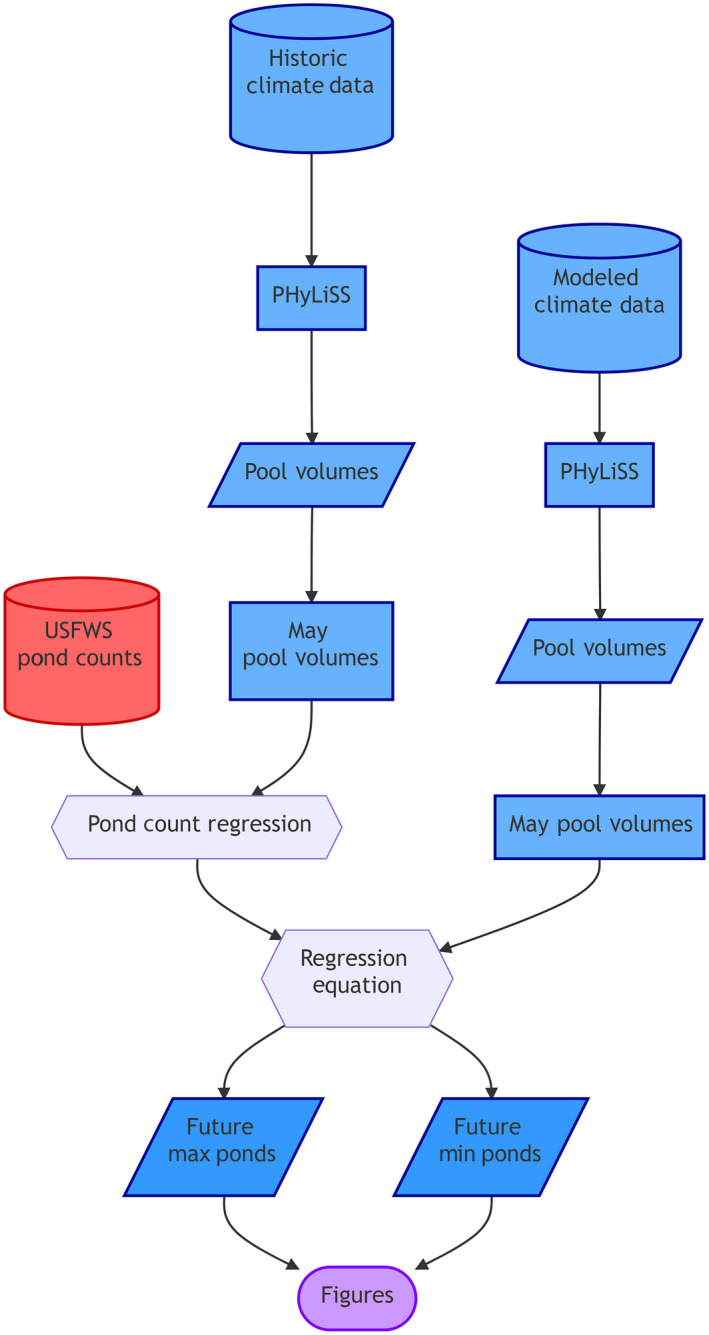
Flow chart for general study design. Meteorological data acquired from Daymet (historical) and coupled model intercomparison project phase 5 (CMIP5; future) databases provide input variables that are processed in PHyLiSS. Pool‐volume outputs are subset to May. Pond‐count data from U.S. Fish and Wildlife Service database and historical May pool volumes are used to prepare a unique regression equation for each site. Modeled future May pool volumes are input into the equations to calculate future May pond‐count outputs from which final visual products are created.

We developed a workflow similar to one described by Sofaer et al. (2017) for using historical and future climate data streams (Fig. 2). First, we calculated mean annual precipitation and temperature for a recent ˜30‐yr period (1982–2015). Historical daily surface temperature and precipitation data were acquired from the 1‐km resolution Daymet data set for our 18 study sites across the PPR. Daymet data are long‐term, continuous, gridded estimates of daily weather and climatology variables by interpolating and extrapolating ground‐based observations through statistical modeling techniques (Thornton et al. 2018; data *available online*).^2^ Historical and future GCM‐generated precipitation and temperature data were acquired from 32 different GCMs (Appendix S1: Table S1) included in the CMIP5 collection of GCMs (Taylor et al. 2012). These data were downscaled to the 1‐km^2^ pixel that each of the 18 study sites resides in using localized constructed analogs (LOCA; Pierce et al. 2014). The LOCA technique uses a multi‐scale spatial matching approach, without averaging, to construct the final analog day chosen from 30 observed days in the historical record with the smallest root mean square error between the observed day and the model day being downscaled without relying on weighted averaging. Overall, this provides a more realistic representation of daily precipitation and temperature than other statistical downscaling methods (Guirguis et al. 2018). The data were acquired from the Bureau of Reclamation climate data archive (see *Open research*) for two 30‐yr periods; one historical (1989–2018) and one at the end of this century (2070–2099).

We used the delta method (Sofaer et al. 2017) to estimate future changes in precipitation and temperature. The delta method adjusts a fine‐scale historical climate data set by shifting the historical values according to the mean change between the 1989–2018 and 2070–2099 periods, as projected by each GCM. This method has been determined most suitable for assessing projected changes in mean climatic conditions (Sofaer et al. 2017). We focused our analysis on annual precipitation and temperature, because wetland ponding in the PPR responds to precipitation and temperature changes on an annual scale (McKenna et al. 2017).

### Mechanistic modeling of wetland hydrology

We simulated wetland pool volume using the PHyLiSS model for a generalized Class II/III (Stewart and Kantrud 1971), seasonally or temporarily ponded wetland for each of the six ecoregions in the United States PPR (Table 1). These wetlands make up an estimated 90% of all intact, prairie‐pothole wetlands (Dahl 2014). The majority of the wetlands sampled by Gleason and Tangen (2008) were embedded within grassland uplands, so all of the generalized class III wetlands in our study also had grassland catchments. PHyLiSS is a mechanistic model that has been used to explore the effects of climate and land‐use change on depressional wetland ecosystems (McKenna et al. 2019, 2021). PHyLiSS was developed using Stella Professional (v. 1.4) software and is publicly available (McKenna et al. 2018). Surface‐water inputs in the model include precipitation, runoff from upland areas of the wetland catchment, and spring snowmelt from snow accumulated through the winter in wetland basins and catchments. Outputs from the wetland include evaporation, transpiration, and overland flow to and from other wetlands when the pool volumes reach a spill point elevation. Subsurface water is also simulated in PHyLiSS as a simplification of complex groundwater dynamics that allows a wetland to continue drying even after the surface pool volume has been completely depleted.

**Table 1:** Mean class II/III wetland and upland catchment area and sample size from the Gleason and Tangen (2008) survey for each of the six ecoregions of the North American Prairie Pothole Region as displayed in Fig. 1.

Ecoregion	Mean wetland basin area (ha)	Mean upland catchment area (ha)	Number of wetland catchments sampled
Canadian aspen forests and parklands	0.32	0.99	11
Central tall grasslands	0.53	1.07	56
Northern mixed grasslands	0.55	1.35	99
Northern short grasslands	0.50	1.47	117
Northern tall grasslands	0.41	1.14	18
Prairie Coteau	0.85	1.74	12

Precipitation in the form of rain either falls directly on the wetland or the upland with surface runoff being calculated with the Soil Conservation Service runoff curve number (CN) method (Cronshey et al. 1986). A “dry, normal, or wet” CN for the given land cover is used to calculate runoff depending on the total 5‐d cumulative precipitation. When the antecedent precipitation is <3.5 cm, the “dry” CN is used, when the antecedent precipitation is between 3.5 and 5.0 cm, the “normal” CN is used, and when the antecedent precipitation is >5.0 cm, the “wet” CN is used. When air temperature is lower than 0°C, precipitation accumulates as snow, which melts when the 10‐d mean air temperature is above 2°C. Snow that accumulates over the wetland area goes directly into ponded water, while the fraction of meltwater from upland snow that enters the wetland as runoff is dependent on the antecedent soil moisture in October of the preceding year as represented by the Palmer Hydrologic Drought Index (PHDI; Palmer 1965). Shallow groundwater losses are accounted for using the equation from Huang et al. (2013). Spill from the basin occurs when the water level of the wetland pond reaches a low point in the divide between adjacent catchments. We have assumed that our generalized wetlands do not receive overland flow inputs from any nearby wetlands. More detailed descriptions and validations of the PHyLiSS model can be found in McKenna et al. (2018).

The ponds of Class II/III, seasonally or temporarily ponded wetlands usually dry by midsummer and have shallow‐marsh vegetation that dominates their central zone (Stewart and Kantrud 1971). Thus, we assumed that climate‐driven changes to subsurface and surface water volumes in this wetland class would be most representative of changes in ponded wetland numbers on a given area of the landscape. This approach has been validated by McKenna et al. (2021) for wetlands and ponded wetland basins for southern North Dakota, USA.

We ran PHyLiSS on a daily time step using historical Daymet (Thornton et al. 2018) precipitation and temperature inputs, and historical and future LOCA downscaled GCM precipitation and temperature data, described in the previous section, as well as ecoregion‐specific wetland bathymetry and upland catchment areas. Wetland area‐to‐volume ratio, maximum wetland area, and catchment area were derived from a sampling of 475 depressional wetlands throughout the U.S. portion of the PPR (Gleason and Tangen 2008). We then calculated ecoregion‐specific relationships for the six PPR ecoregions (Fig. 1; Table 1). PHyLiSS code is publicly *available online*.^3^

PHDI is a climatic variable that is used in PHyLiSS to estimate snowmelt dynamics. The antecedent October monthly PHDI determines the proportion of snowpack that melts and is added to the wetland basin as runoff. The higher antecedent PHDI increases the probability of a “frost seal” that allows for more spring snowmelt runoff to be added to the wetland basin water budget rather than be evaporated or infiltrated in the upland (Fang and Pomeroy 2007). In order to calculate PHDI using future precipitation and temperature data, and soil available water‐holding capacity we used a tool developed in the program MATLAB (Jacobi et al. 2013). This tool uses an algorithm to replicate PHDI calculations published by the National Oceanographic and Atmospheric Administration (NOAA 2016). We compared historical MATLAB to NOAA monthly PHDI values (1982–2015) and used the regression models to adjust future MATLAB‐generated PHDI inputs to PHyLiSS (Appendix S1: Fig. S2).

### Modeling climate‐driven changes in hydrology of waterfowl habitat

After running PHyLiSS daily from 1982 to 2015, we summarized wetland volumes (both surface and subsurface water) in May to estimate the relationship between PHyLiSS output for one typical temporarily ponded wetland and the total number of May ponds in each USFWS survey stratum (Dahl 2014). These field‐based May pond counts have formed the basis of much prior work on waterfowl population trends in the PPR (Niemuth and Solberg 2003). We also used supplemental data from the Minnesota Department of Natural Resources to better enhance our May pond‐count data set (MNDNR 2019). We then used simple linear regression to determine the best‐fit relationship between PHyLiSS simulated water volumes and empirical May pond counts.

To create a baseline for number of May ponds, we compared observed historical climate data (1980–2018) to USFWS May pond‐count data for each stratum in which the selected sites reside. We used historical precipitation, temperature, and PHDI PHyLiSS inputs during the same time period to generate wetland pool volumes, then we extracted May pool volumes for the years 1982–2015. We used simple linear regression between modeled pool volumes and USFWS pond counts to create a unique equation for each site describing this relationship (Appendix S1: Table S2; Fig. S1).

Average May pond percent change was calculated by estimating percent change between two 30‐yr climate periods (1989–2018 and 2070–2099) from May pond estimates generated using GCM inputs. The percent change from historical to future climate period was then applied to the observed May pond‐count estimates from historical precipitation and temperature data (1982–2015) to estimate the increase or decrease in the number of May ponds, i.e., the delta method (Sofaer et al. 2017).

We selected the wettest and driest future climate models with the largest and smallest numbers of May ponds (Appendix S1: Table S3) to develop a Hot‐Wet and Hot‐Dry scenario estimates that included the possible range of future, landscape‐scale, May pond numbers (2070–2099). We used the SAGA thin plate spline interpolation geoalgorithm in QGIS v. 3.6 (Conrad et al. 2015) to estimate May pond density (no./km^2^) across a continuous matrix between our study points. The thin plate spline is a geometric function that “bends” the spatial arrangement to adjust to the values of the points (Garza‐Gisholt et al. 2014).

## Results

**Fig. 3:**
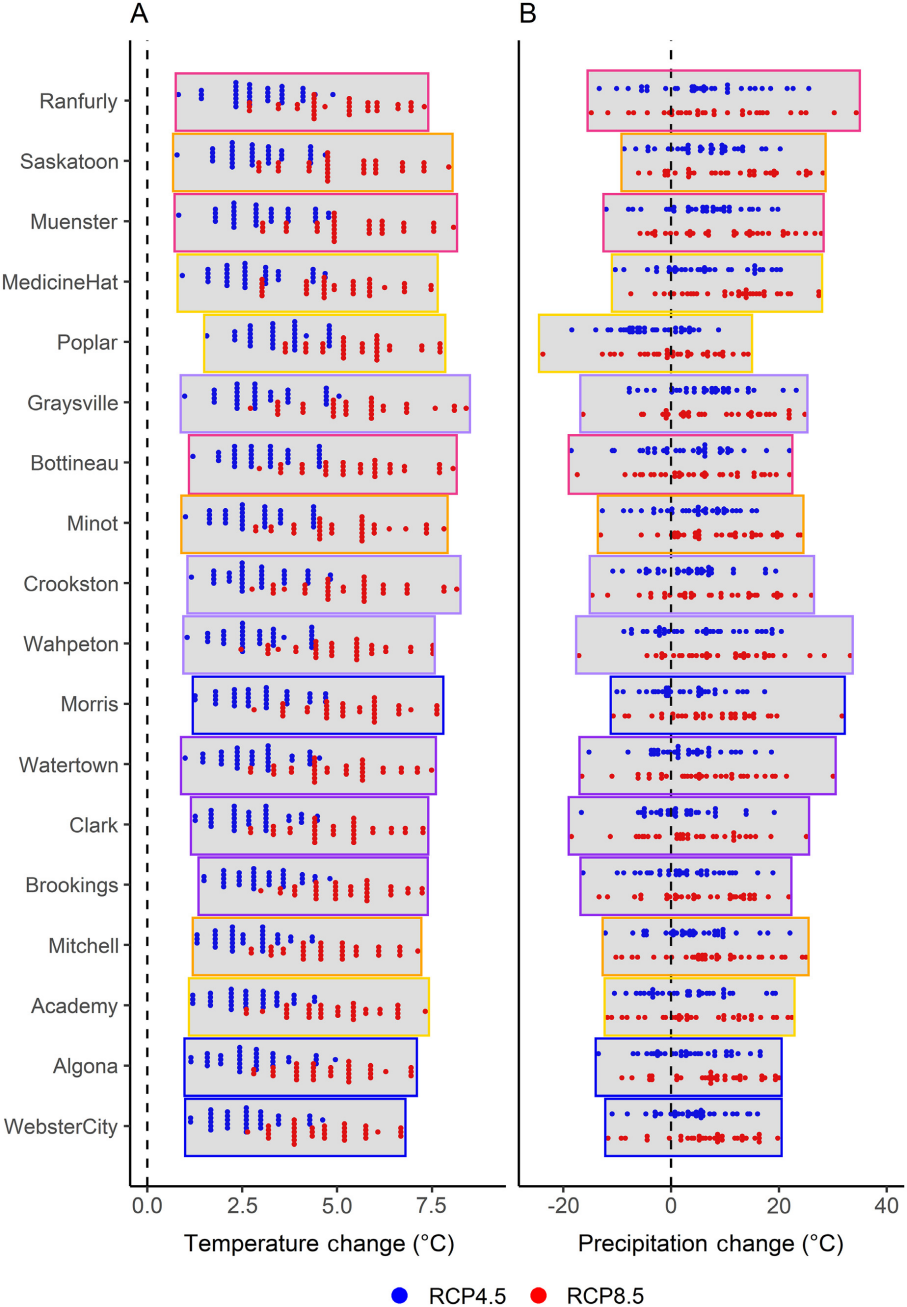
Climate‐change projections for coupled model intercomparison project phase 5 (CMIP5) models under low emission scenario RCP 4.5 (blue dots) and high emission scenario RCP 8.5 (red dots). Panel A shows the change (°C) in 30‐yr mean annual temperature and Panel B shows percent change in mean annual precipitation from 1989–2018 to 2070–2099. Vertical dashed lines represent no change. Eighteen study sites in the North American Prairie Pothole Region are represented from north to south and bounding boxes are color coded by ecoregion.

**Fig. 4:**
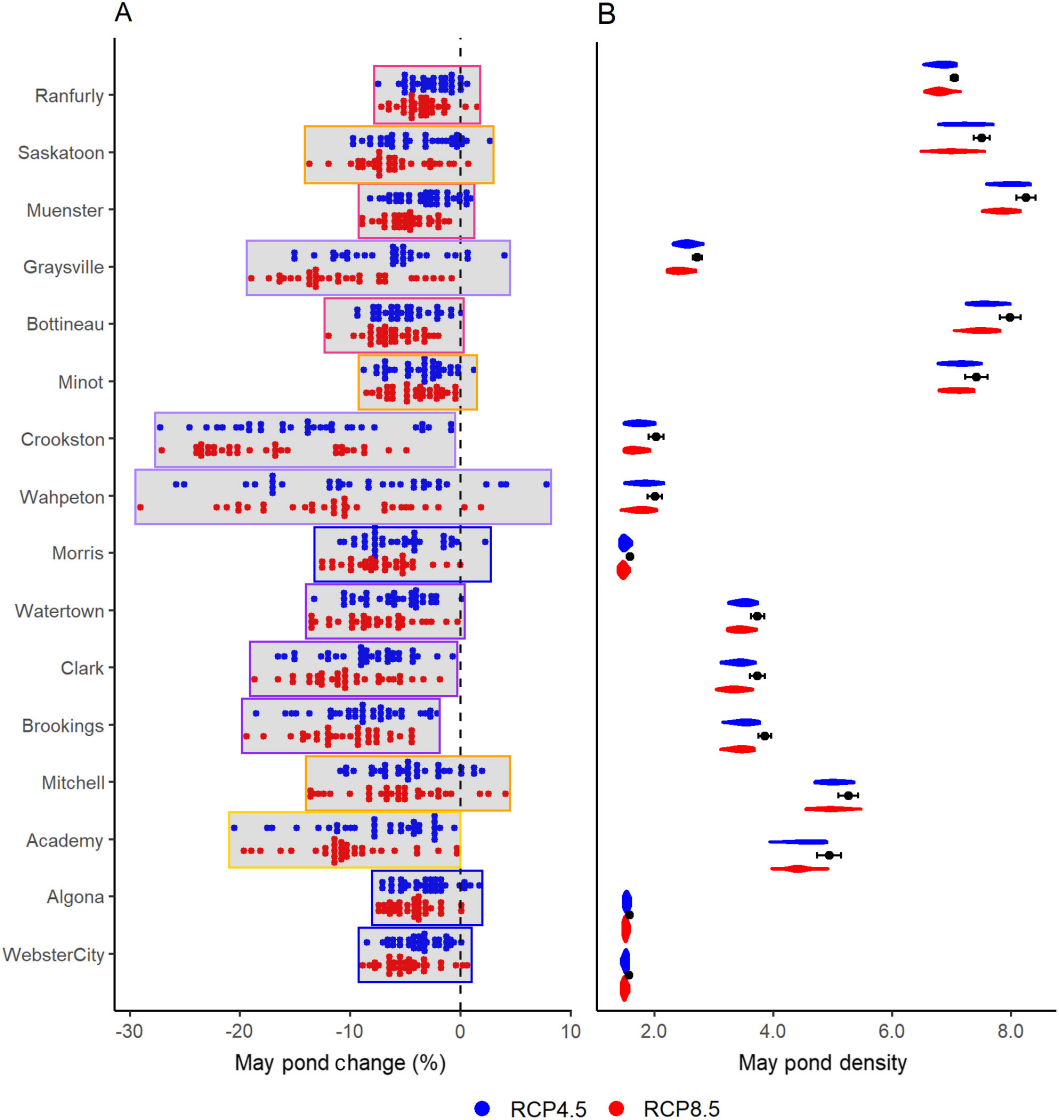
Projected average changes in May pond counts from 1989–2018 (black dotted line) to 2070–2099 for future coupled model intercomparison project phase 5 (CMIP5) climate models under low emissions scenario RCP 4.5 (blue dots) and high emissions scenario RCP 8.5 (red dots) in the North American Prairie Pothole Region. Each dot represents a change in May ponds under a unique climate future. Panel A shows percent change in May ponds with the vertical dashed line representing no change. Panel B shows the number of May ponds for each site. The black dots with standard error bars represent historical mean number of May ponds and the RCP 4.5 and RCP 8.5 model projections are represented by the blue and red violin symbols for each site in Panel B. The shape of the violin represents the clustering of the different May pond projections under all the unique climate futures. Matching colored boundaries indicate stations are within the same ecoregion.

Overall, we found all the climate models predicted hotter mean annual temperature and three‐quarters of the models predicted increases in mean annual precipitation at the end of the century (2069–2099) compared to historical averages (1989–2018; Fig. 3). Temperature and precipitation vary across a gradient in the PPR with the northwestern region historically the driest, and the southeastern region historically the wettest. This pattern remained the same in each of our scenario runs, although at varying degrees of intensity. We found that, depending on the location in the PPR, the range of future precipitation and temperature possibilities could fall anywhere between a 29% loss and an 8% increase in the number of May ponds (Fig. 4).

**Fig. 5:**
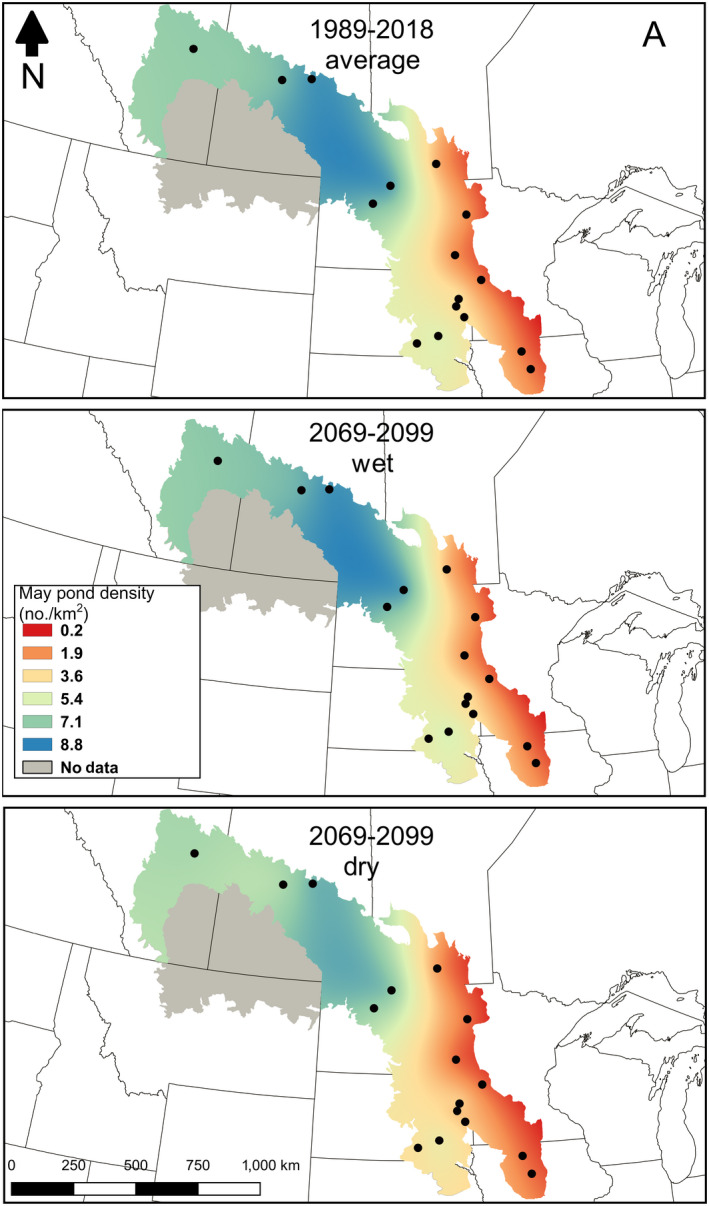
Map of wetland pond density under three scenarios. Panel A is the historical 30‐yr (1989–2018) mean May pond count interpolated between the downscaled pond density of 15 study sites. Panel B and C are both future 30‐yr (2070–2099) mean May pond counts interpolated between the downscaled pond density of 16 study sites. (B) The Wet‐Hot Future had the highest May pond estimate from the future climate models and (C) the Dry‐Hot Future had the lowest May pond estimate from the future climate models.

In 16 of our 18 sites we found statistically significant relationships between PHyLiSS May pool volume and the May pond density for the corresponding year 1982–2015 with mean *R* ^2^ of 0.40 for all significant models (Appendix S1: Fig. S1; Table S2). Results for the northwestern‐most region of the PPR including areas of Montana and Alberta lacked statistical significance between PHyLiSS model output and May ponds therefore we excluded these stations from analysis (Figs. 4, 5). Under a Hot and Wet future, May ponds were predicted to increase by 1%, however in a Hot and Dry future May ponds were predicted to decrease by 12%. In both the Hot‐Wet and Hot‐Dry futures, the distribution of May ponds was not predicted to change significantly, i.e., May ponds remained most heavily concentrated in the north‐central portion of the PPR (Fig. 5).

### Projected changes in precipitation and temperature throughout the PPR

All models for each emissions scenario projected an increase in average temperature between 0.8–8.4°C (Fig. 3A). Models under emissions scenario RCP4.5 had average temperature increases between 0.8°C and 5.1°C, where more extreme temperature changes occurred under emissions scenario RCP8.5 increasing on average between 2.5°C and 8.4°C. Projected changes in average precipitation varied between models and emissions scenarios, however, nearly three‐quarters of the models predicted increases in precipitation (Fig. 3B). Under both emissions scenarios, changes in precipitation ranged from −18.5% to +33.2%.

### Climate‐driven shifts in ponding of prairie pothole wetlands

The percent change in May pond density for individual weather stations ranged from −73% to +20%. When compared to the 30‐yr historical average, these changes represent an overall 6% increase in May ponds under the Hot‐Wet future, and a 17% decrease under the Hot‐Dry future (Fig. 4A). Historically, the northwestern portion of the PPR had the highest densities of May ponds and this pattern was maintained under both the Hot‐Wet and Hot‐Dry scenarios. We estimated the average number of May ponds historically to be approximately 3.60 million. Under the wettest and driest possible futures, we estimated an occurrence of between 3.65 and 3.17 million May ponds, respectively (Fig. 5).

Under the Hot‐Wet future (Fig. 5B), north central Saskatchewan had the greatest density of May ponds (˜8 ponds/km^2^) along with northwestern North Dakota (˜7 ponds/km^2^). Areas around southeastern North Dakota and northwestern Minnesota contained lower May pond densities (˜3 ponds/km^2^) while much of the southern region of the PPR was moderately dry (˜5 ponds/km^2^). In the Hot‐Dry future, the wet and dry areas remained the same in relation to each other, although they were much drier overall (Fig. 5C). The northern region of the PPR was moderately wet (˜6 ponds/km^2^), however the drought conditions increased substantially in already dry areas of the eastern and southern regions (˜1–3 ponds/km^2^).

## Discussion

Our results suggest that current management and conservation strategies for wetlands in the PPR that focus on areas with the highest densities of intact wetland basins (Niemuth et al. 2014) and that support large numbers of breeding duck pairs will likely also be the most successful in maintaining habitats critical to continental waterfowl populations under an uncertain climate future. These results support some modeling studies that suggest a minimal shift in the future distribution of May ponds in the PPR (Sofaer et al. 2016, McIntyre et al. 2019) and are in contrast to other studies that have suggested a large, southeastern shift in viable waterfowl habitat in the PPR (Johnson et al. 2005, Johnson and Poiani 2016). The most likely reason our results differed from Johnson and Poiani (2016) is a combination of our modeling approach, using May pond data to identify a more realistic distribution of wetland basins throughout the PPR, and using a more robust and up to date suite of future climate models.

Our projections can be continually improved to include additional climate dynamics like relationships developed by Abel et al. (2020) that include the response of May ponds to sea surface temperature oscillations and large atmospheric‐pressure anomalies. The impact of climate change on increasing the frequency of high‐intensity storms could continue the shift from snowmelt‐driven wetland hydrology (Shook and Pomeroy 2012) to summer and fall precipitation‐driven wetland hydrology that has already been observed in the last 25‐yr in the southern PPR (McKenna et al. 2017). Sustained wet periods under increased‐precipitation climate scenarios can result in a change in the hydroperiods of small wetlands resulting in these wetlands remaining wetter longer and drying less frequently. Small wetlands are known to provide disproportionate contributions to hydrologic, biogeochemical, and ecological functions than would be predicted by their proportional area in any given watershed (Calhoun et al. 2017). Thus, a loss of smaller wetlands and convergence towards more homogeneous wet/dry cycling can limit the diversity of species that use wetlands of the PPR (McLean et al. 2019).

A similar loss of smaller wetlands can occur due to prolonged dry periods with larger wetlands becoming smaller in volume on average and supporting different plant and macroinvertebrate communities along what is known as the “wetland continuum” (Euliss et al. 2004, Mushet et al. 2018). Prolonged dry periods have also been known to change the groundwater hydrology of some prairie‐pothole wetlands where water tables drop and salts can be flushed from dry wetlands during rainfall events (Levy et al. 2018). These changes in salinity can also influence the plant and macroinvertebrate communities. There is evidence that upland management practices can increase water inputs during dry years, especially when precipitation events are delivered more frequently as extreme events. Both grazing and burning can reduce the vegetative structure that could lead to increases in runoff in grassland catchments thereby reducing pond losses during the waterfowl breeding season (McKenna et al. 2021).

These shifts in timing of precipitation and wetland ponding can also have impacts on the spatial and temporal synchrony between migratory animals and their available habitat (Koenig and Liebhold 2016). For long‐distance migrants, climate change might advance the phenology of their breeding areas, but their endogenous migration triggers might not be in sync with these changes (Both and Visser 2001). Alterations in migration dynamics for birds related to atmospheric warming have already been documented. In the eastern United States, average arrival dates of 32 North American passerine species have occurred significantly earlier over time due to increased temperatures (Miller‐Rushing et al. 2008). Atmospheric warming in Québec, Canada has been shown to cause Greater Snow Geese (*Chen caerulescens atlantica)* to arrive in Arctic breeding grounds earlier and stay longer causing greater stress on wetland‐vegetation communities (Gauthier et al. 2005). These shifts might impact the current management paradigm that correlates the number of breeding birds in the PPR to the number of May ponds. If the timing of breeding shifts to different months, then the importance of ponded wetlands for those birds will also shift. For example, April or June ponds might be a better predictor of breeding waterbird numbers in the future.

Our study was designed intentionally to both advance the scientific understanding of the responses of wetland hydrology to climate change and provide information that can be effectively integrated in strategic landscape‐scale planning for the acquisition, restoration, and management of wetlands. Sofaer et al. (2017) emphasized the importance of describing and justifying methodologies for incorporating climate‐change projections to ecological studies. Similar scrutiny should be taken when selecting wetland‐modeling approaches. Wetland‐hydrology models that rely heavily on generalized statistical relationships could be greatly improved by incorporating a mechanistic understanding of the complex relationships between climate variables and wetland ecohydrological functioning. Ultimately, when these precautions and best practices are implemented, we improve the accuracy of projections and the likelihood that findings can be used directly by the management community.

## Supporting information

Appendix S1

## Data Availability

Data are archived with the US Geological Survey North Central Climate Adaptation Science Center at https://www.sciencebase.gov/catalog/item/5b33be6fe4b040769c172fad. The Pothole Hydrology Linked Systems Simulator (PHyLiSS) model code (McKenna et al. 2018) is available in the USGS ScienceBase Catalog: https://doi.org/10.3133/ofr20181165. Modeled climate data are publicly available through the US Bureau of Reclamation climate data archive: https://www.sciencebase.gov/catalog/item/551973bfe4b0323842783127.

## Footnotes

^2^

https://daymet.ornl.gov/

^3^

https://www.sciencebase.gov/catalog/item/5b840f3ee4b05f6e321b4f04
