# Supplementary material for: Limited shifts in the distribution of migratory bird breeding habitat density in response to future changes in climate
Source: Ecol Appl. 2021 Aug 30;31(7):e02428. doi: 10.1002/eap.2428 (PMC9285366; doi:10.1002/eap.2428)
Supplement: Supplementary file 1 — Appendix S1 [file EAP-31-0-s001.pdf]

Supporting Information. McKenna, O.P, D.M. Mushet, S.R. Kucia, and E.M. McCulloch-Huseby. 2021. Limited shifts in the distribution of migratory bird breeding habitat density in response to future changes in climate. Ecological Applications.

Appendix S1

Table S1. Coupled model intercomparison project phase 5 (CMIP5) models (Taylor et al. 2012) list

Model	Model	Model
access1-0	csiro-mk3-6-0	inmcm4
access1-3	ec-earth	ipsl-cm5a-lr
bcc-csm1-1	fgoals-g2	ipsl-cm5a-mr
bcc-csm1-1-m	gfdl-cm3	miroc-esm
canesm2	gfdl-esm2g	miroc-esm-chem
ccsm4	gfdl-esm2m	miroc5
cesm1-bgc	giss-e2-h	mpi-esm-lr
cesm1-cam5	giss-e2-r	mpi-esm-mr
cmcc-cm	hadgem2-ao	mri-cgcm3
cmcc-cms	hadgem2-cc r	noresm1-m
cnrm-cm5	hadgem2-es	

Literature Cited

Taylor, K. E., R. J. Stouffer, and G. A. Meehl. 2012. An Overview of CMIP5 and the Experiment Design. Bulletin of the American Meteorological Society 93:485-498.

Table S2. Regression analysis statistical relationships between May pool volumes and calculated May-pond counts in the North American Prairie Pothole Region, visualized in Figure S1.

Weather station	May Pool Volume:May Pond Count R^2 (1981-2015)	P-value
Ranfurly	0.33	0.0004
Saskatoon	0.55	0.0000005
Muenster	0.23	0.005
Medicine Hat	0.06	0.15
Poplar	0.14	0.09
Graysville	0.24	0.004
Bottineau	0.49	0.000005
Minot	0.28	0.001
Crookston	0.41	0.00004
Wahpeton	0.47	0.00002
Morris	0.62	0.00000003
Watertown	0.30	0.004
Clark	0.37	0.0009
Brookings	0.33	0.001
Mitchell	0.40	0.00006
Academy	0.60	0.0001
Algona	0.37	0.0001
Webster City	0.28	0.001

Table S3. Global Climate Models used for each site examined in the North American Prairie Pothole Region for constructing heat maps (Figure 5).

Site	Highest May Ponds		Lowest May Ponds	
	Emission Scenario	Model	Emission Scenario	Model
Academy	RCP4.5	cesm1-bgc	RCP8.5	miroc-esm
Algona	RCP4.5	gfdl-esm2m	RCP8.5	miroc-esm
Bottineau	RCP4.5	gfdl-esm2g	RCP8.5	access1-0
Brookings	RCP8.5	gfdl-esm2g	RCP8.5	giss-e2-h
Clark	RCP4.5	gfdl-esm2g	RCP8.5	ipsl-cm5a-lr
Crookston	RCP8.5	gdfl-cm3	RCP8.5	ipsl-cm5a-lr
Graysville	RCP4.5	gfdl-esm2g	RCP8.5	ipsl-cm5a-lr
Minot	RCP4.5	gfdl-esm2g	RCP8.5	giss-e2-r
Mitchell	RCP4.5	hadgem2-cc	RCP8.5	miroc-esm
Morris	RCP4.5	cmcc-cms	RCP4.5	access1-0
Muenster	RCP4.5	gfdl-esm2g	RCP4.5	access1-0
Saskatoon	RCP4.5	Csiro-mk3-6-0	RCP8.5	ipsl-cm5a-mr
Wahpeton	RCP4.5	gdfl-cm3	RCP8.5	hadgem2-es
Watertown	RCP4.5	gfdl-esm2g	RCP8.5	miroc-esm
Webstercity	RCP4.5	gfdl-esm2g	RCP8.5	miroc-esm

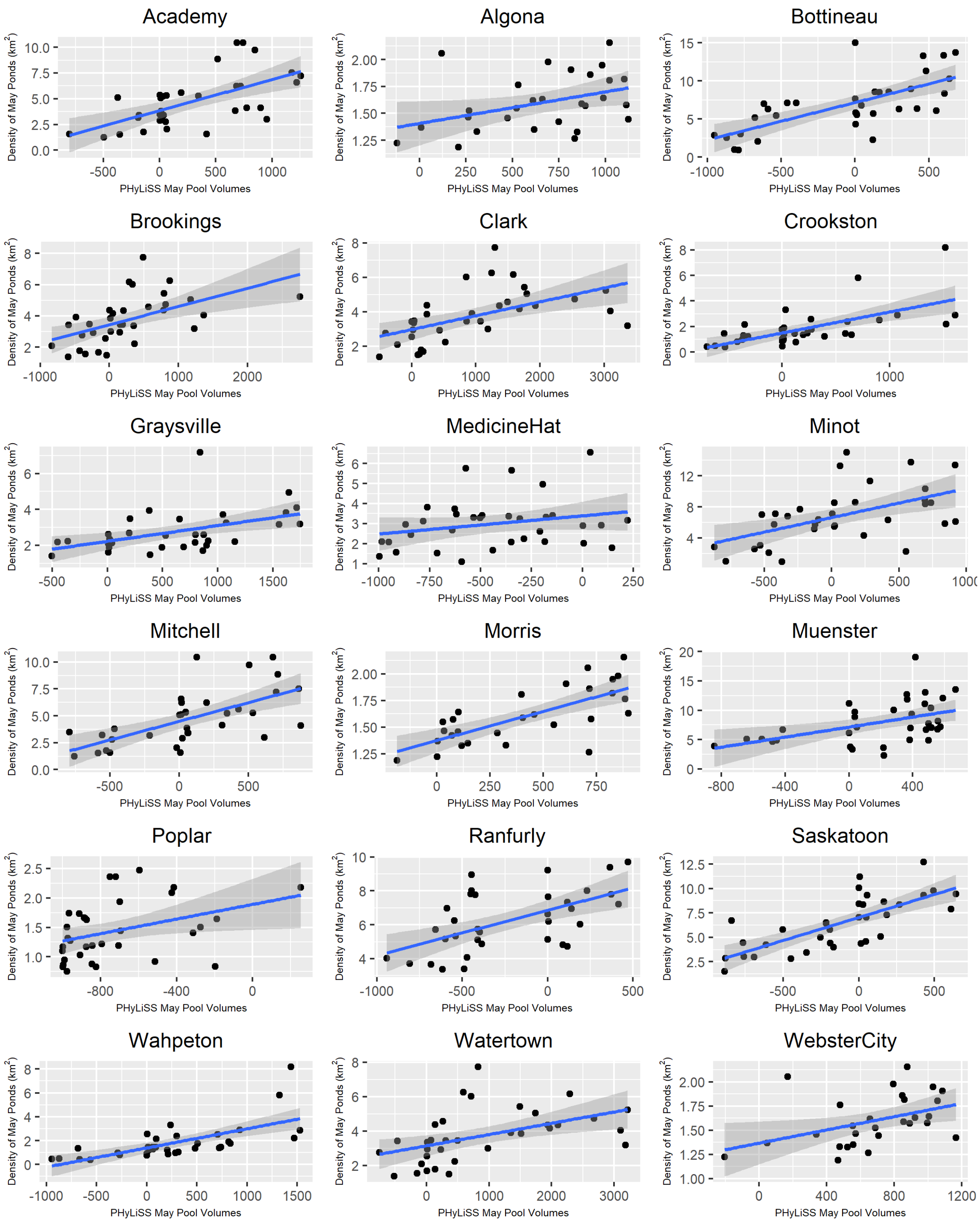

Figure S1. Best-fit linear regression models for estimating relationships between PHyLiSS May pool volumes and Regional May pond counts (1982–2015) in the North American Prairie Pothole Region. All models except Poplar and Medicine Hat models are significant ($p < 0.05$).

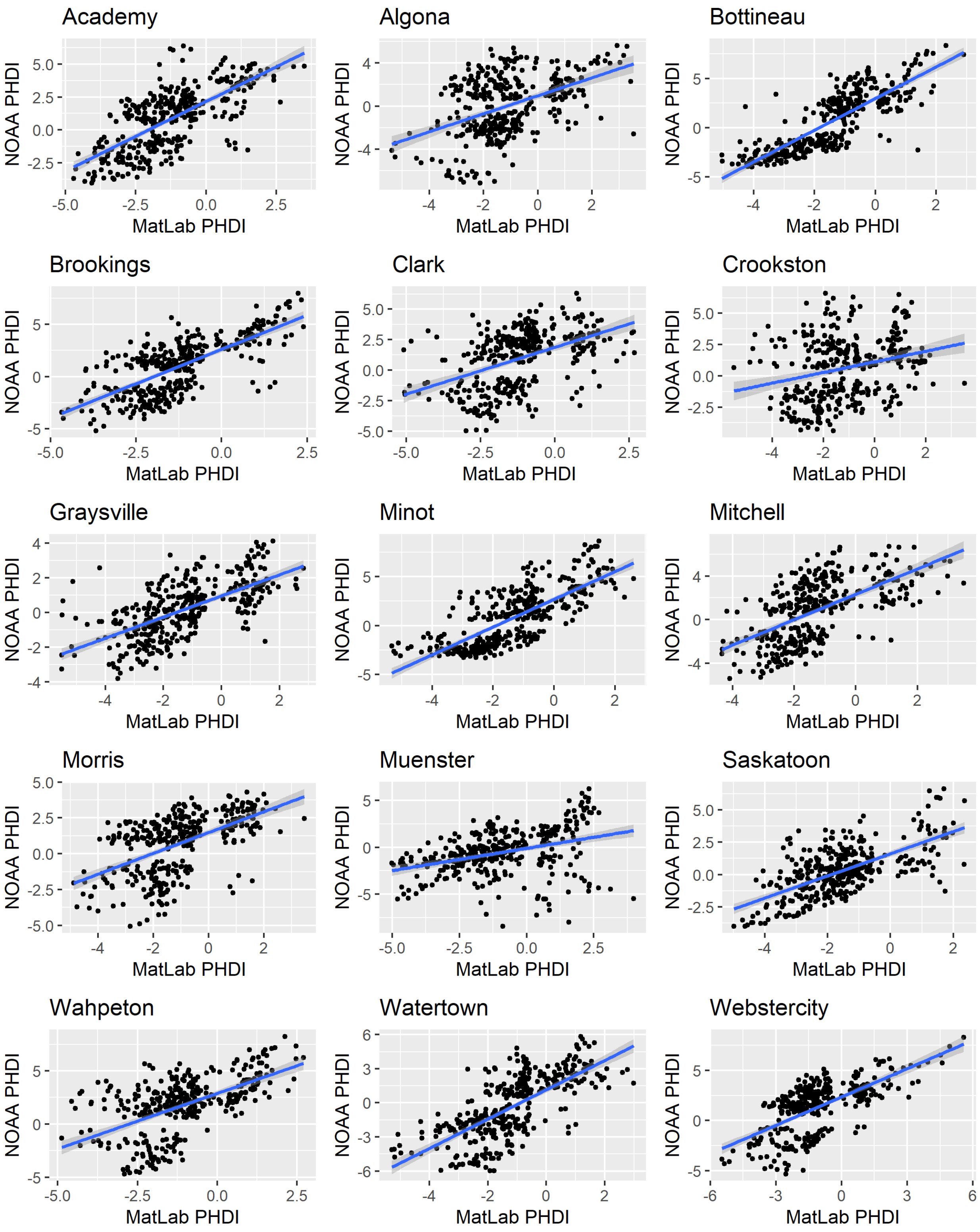

Figure S2. Linear models visualizing relationships between published National Oceanographic and Atmospheric Administration (NOAA) monthly Palmer Hydrologic Drought Index (PHDI) values and MatLab-developled monthly PHDI estimate for each of the 18 study sites in the North American Prairie Pothole Region (1982–2015).
